# An Unusual Presentation of Parvimonas micra Infective Endocarditis

**DOI:** 10.7759/cureus.3447

**Published:** 2018-10-13

**Authors:** Dawn Ho, Grace Ang, Chaozer Er, Siew Fei Yap, Veeraraghavan Meyyur Aravamudan

**Affiliations:** 1 Internal Medicine, MOH Holdings, Singapore, SGP; 2 Internal Medicine, Woodlands Health Campus, Singapore, SGP; 3 Cardiology, Yishun Health, Singapore, SGP; 4 Internal Medicine, Woodlands Health Care, Singapore, SGP

**Keywords:** parvimonas micra, endocarditis, peptostreptococcus, anaerobic bacteria

## Abstract

Parvimonas micra has been identified as a prominent oral pathogen. This organism has been implicated in periodontal, soft tissue and bone infections. It causes a subacute presentation with high morbidity. We present a case of severe infective endocarditis caused by Parvimonas micra requiring valvular surgery despite appropriate antibiotics. To our knowledge, this is the second case report of Parvimonas micra infective endocarditis since its reclassification in 2006.

## Introduction

Parvimonas micra is an anaerobic Gram-positive coccus. Anaerobic bacteria have been linked to severe complications of infective endocarditis (IE) such as mycotic aneurysms, septic emboli, valvular destruction, and septic shock [1]. IE due to anaerobic bacteria is uncommon, accounting for 2–16% of all cases of IE over the past few decades. However, the Parvimonas species in particular is noted in a select group of patients predisposed with prosthetic valves, dental procedures and infections, septic arthritis, or genitourinary infections [1].

In this case, we describe a 42-year-old Malay gentleman with a mitral valve replacement, who presented with recurrent fever after a tooth extraction. He was diagnosed to have Parvimonas micra infective endocarditis.

This is the second case report of Parvimonas micra causing IE in humans since the organism, originally known as Peptostreptococcus micros, was transferred to the Micromonas genus in 1999 [2], then reclassified within the Parvimonas genus in 2006 [2, 3]. We reviewed the literature in the context of this uncommon infection. We included in our literature review species formally classified as Peptostreptococcus species.

## Case presentation

A 42-year-old Malay gentleman, with significant past medical history of diabetes mellitus and mechanical mitral valve replacement (MVR) (St. Jude Medical Masters 29 mm) due to Staphylococcal endocarditis in 2015, on lifelong anticoagulation with warfarin, was first admitted for a one-week history of fever. Two days before his admission, he had a molar tooth extraction, and was given amoxicillin postoperatively for dental prophylaxis. He was not given any antibiotic prophylaxis before his tooth extraction. Other than fever, chills and cough, he had no other infective symptoms.

He also had significant bleeding of the tooth extraction wound causing symptomatic anemia with giddiness and dyspnea.

Physical examination revealed pyrexia (temperature of 38.6℃) and stable haemodynamics. His heart sounds were crisp with a metallic first heart sound. There were no peripheral stigmata of IE.

Routine investigations showed slightly elevated inflammatory markers and severe anemia (Table 1). The chest radiograph did not reveal any opacities suggestive of septic embolism (Table 1). International normalized ratio (INR) was found to be 4.99 (latest INR before admission: 2.9). Two sets of blood culture showed no bacterial growth (Table 2). Transthoracic echocardiogram (TTE) and transoesophageal echocardiogram (TOE) were not done.

**Table 1:** Trend of inflammatory markers and other investigations. MCV: Mean corpuscular volume; MCH: Mean corpuscular hemoglobin; MCHC: Mean corpuscular hemoglobin concentration; MPV: Mean platelet volume; RDW: Red cell distribution width.

Test	Results					Unit	Reference Level
	First admission	First admission (Discharge)	Second admission	Second admission (Discharge)	Third admission		
White blood cells (WBC)	19.79	12.98	13.99	11.88	11.06	10^9^/L	3.37-8.38
Red blood cells (RBC)	2.46	2.74	3.45	3.40	3.66	10^12^/L	4.29-5.70
Hemoglobin (Hb)	6.8 (Baseline Hb: 8-10)	7.4	9.3	8.4	9.0	g/dL	13.3-16.6
Hematocrit	19.9	23.1	28.5	27.1	28.7	%	41.3-52.1
MCV	80.9	84.3	82.6	79.7	78.4	fL	86.7-102.3
MCH	27.6	27	27	24.7	24.6	pg	27.1-32.4
MCHC	34.2	32	32.6	31	31.4	g/dL	29.7-33.1
Platelets	315	249	348	385	424	10^9^/L	172-378
MPV	9.6	9.2	9.4	9.4	9.4	fL	9.2-12.0
RDW	14.8	15.3	15.2	15.4	15.1	%	12.2-14.8
Sodium	135	137	140	138	139	mmol/L	135-145
Potassium	4.5	4	4.1	3.9	4.1	mmol/L	3.5-5.1
Chloride	99	100	103	102	101	mmolL	96-108
Bicarbonate	22	23	23	23	23	mmol/L	23-29
Creatinine	111	107	114	125	119	umol/L	59-104
Urea	8.6	6.2	4.7	2.3	4.8	mmol/L	2.8-7.6
Anion gap	17.9	18.6	17.8	17.1	19.0		8-16
Albumin	30		37		40	g/L	35-50
Bilirubin, total	7		14		17	umol/L	3-21
Alanine aminotransferase	17		27		19	u/L	10-44
Aspartate aminotransferase	17		18		20	u/L	10-34
Alkaline phosphatase	59		74		75	u/L	45-122
Gamma-glutamyl transferase	81		113		73	u/L	11-50
C-reactive protein	16.6		52	15.1	10.2	mg/L	1-5
Procalcitonin	0.22		0.15			ng/mL	0.5-2
Chest radiograph	No consolidation, pleural effusion or pulmonary embolism						

**Table 2:** Blood cultures during the three admissions.

Admission (Date)	First admission (19/3/18)	Second admission (1/4/18)	Second admission (2/4/18)	Second admission (11/4/18)
Blood culture results	Two sets negative	Two sets negative	First and second sets grew Parvimonas micra from anaerobic culture	Two sets negative

He was given 4 units of packed cell transfusions for his symptomatic anemia and oral vitamin K for warfarin reversal. Adrenaline gauze packing and tranexamic acid gargle were given for hemostasis of the tooth extraction wound. He was started on amoxicillin/clavulanic acid for dental prophylaxis. He remained afebrile in the ward and was discharged. INR before discharge was 2.32.

**Figure 1:**
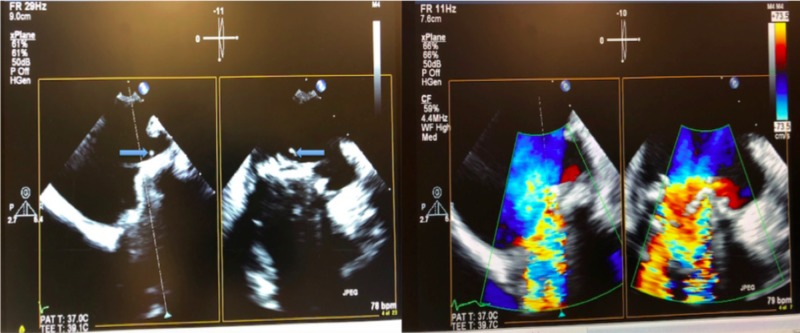
(Left) Mobile friable vegetations arising from the mitral prosthetic valve. (Right) Severe mitral regurgitation with torrential back flow across the prosthetic mitral valve demonstrated by Colour Doppler.

One week later, he was admitted for another episode of fever. This time, the first two sets of blood culture again did not reveal any bacterial growth. However, the third and fourth sets of blood culture were positive and grew Parvimonas micra (Table 2). TTE showed a stable MVR with no vegetation and normal function of the MVR. However, TOE showed that the MVR had at least two vegetations, with a new severe mitral regurgitation (MR) (Figure 1).

He was referred to the cardiologist and the infectious disease (ID) specialist. Initial antibiotic treatment was ceftriaxone and vancomycin. After two repeated sets of negative blood culture (Table 2), antibiotic was switched to penicillin G with the intention to complete a six-week course.

However, one month later, he was readmitted for exertional dyspnea and orthopnea. He had no fever. Inflammatory markers were unremarkable (Table 1). TTE showed a normal MVR again and was unable to quantify the severity of the MR, but unchanged mean mitral valve pressure gradient (MVPG). TOE further showed shrinkage of vegetations but severe MR, and also increased MVPG.

Penicillin G was continued. He was given diuretic for his heart failure. After consulting the cardiothoracic surgeon, the patient underwent a repair operation of the mitral paravalvular leak. Intraoperative tissue culture showed no bacterial growth. Postoperative blood culture was negative.

He remained stable after repair of his MVR.

## Discussion

Parvimonas micra is part of the normal commensal flora of the gastrointestinal tract and the gingival crevice, and has been implicated in infections of the periodontal area, soft tissue and bone [2]. Prosthetic valves, dental procedures and infections, septic arthritis, and genitourinary infections are predisposing conditions for IE caused by Parvimonas micra [1].

IE caused by anaerobic bacteria remains uncommon but carries a high mortality rate of 21–43% [1]. Mycotic aneurysms are a common complication with anaerobic IE. Other complications include valvular destruction, aortic ring abscess, aortitis, cardiogenic shock, dysrhythmia, and septic shock [1].

The subacute presentation of anaerobic IE can delay the diagnosis [1, 4]. This may explain the high rate of complications and frequent need for surgery, as observed in our case.

Minces et al. noted a high rate of valvular complications in Peptostreptococcus IE [4]. Thirty-five percent of the cases required surgery. The duration of symptoms prior to diagnosis could last as long as an average of seven weeks, and as many as 45% of the cases had persistently negative blood cultures [4]. The subacute presentation of Parvimonas micra IE can lead to delays in diagnostic cardiac imaging [5]. Time to definitive echocardiogram (TTE or TOE) has been found to be an important predictor of valve destruction, embolization, and subsequent valve surgery [5]. In our patient, cardiac imaging was not done during the first admission due to the subtle nature of his presentation in the absence of classical clinical features such as new heart murmurs, embolic complications, or vascular or immunologic phenomena, along with negative blood cultures.

We found that even with improving inflammatory markers (Table 1), negative repeated blood cultures (Table 2), and shrinkage of vegetations, our patient developed severe valve dysfunction requiring surgery. This reveals that Parvimonas micra is aggressive bacteria that can lead to severe valve dysfunction despite appropriate antibiotics. Hence Parvimonas micra IE is an infection that requires closer monitoring. Identification of Parvimonas micra in blood cultures should prompt careful evaluation, particularly considering the subacute presentation, and high morbidity, of this infection.

Repeat echocardiograms including ongoing surveillance echocardiograms, particularly TOEs, are suggested. In our patient, TOE was necessary in the diagnosis of IE while TTE was negative during both the second and third admissions. This is not surprising as the sensitivity for the diagnosis of vegetations in native and prosthetic valves is 70% and 50%, respectively, for TTE and 96% and 92%, respectively, for TOE [6].

Surveillance echocardiogram is not only helpful in monitoring regression of vegetations, but is necessary in detecting new silent complications, including cardiac and valvular morphology and function, especially given the hostile course of Parvimonas micra IE. Young et al. reported a case of Parvimonas micra IE who developed severe native valve endocarditis complicated by aortic valvular destruction and perivalvular abscess, requiring emergent cardiac surgery with a protracted intensive care unit (ICU) course following surgery [7]. Our two cases reiterate the aggressive nature of this infection.

Interestingly, our patient was not given antibiotic prophylaxis before his tooth extraction. In the 2015 European Society of Cardiology (ESC) guidelines [6], antibiotic prophylaxis should only be considered for patients at highest risk for endocarditis, including: 1. Patients with any prosthetic valve, 2. Patients with a previous episode of IE, and 3. Patients with congenital heart disease, who are undergoing dental procedures requiring manipulation of the gingival or periapical region of the teeth or perforation of the oral mucosa, and is not recommended in other situations. The recommended prophylaxis is a single dose of amoxicillin or clindamycin if penicillin-allergic 30–60 minutes before the procedure. Given that prosthetic valves and dental procedures are predisposing conditions for IE caused by Parvimonas micra [1], antibiotic prophylaxis may have prevented this protracted episode of Parvimonas micra IE in our patient.

## Conclusions

In conclusion, our case report demonstrates that Parvimonas micra IE is rare but associated with significant morbidity. In light of the subacute presentation of Parvimonas micra IE, the early use of cardiac imaging (TTE or TOE) despite subtle clinical features and negative blood cultures, with a low threshold for TOE, might be beneficial in a select group of patients with high index of suspicion, namely patients predisposed with prosthetic valves and recent dental procedures.
